# Pharmacotherapy Profiles in People with Opioid Use Disorders: Considerations for Relevant Drug–Drug Interactions with Antiviral Treatments for Hepatitis C

**DOI:** 10.3390/pathogens10060648

**Published:** 2021-05-24

**Authors:** Andreas Hintz, Tim Umland, Gero Niess, Mehtap Guendogdu, Anika Moerner, Frank Tacke

**Affiliations:** 1 Alexander-Apotheke, 20099 Hamburg, Germany; team@alexapo.de (A.H.); umlandtim@gmx.net (T.U.); 2 Gilead Sciences GmbH, 82152 Munich, Germany; gero.niess@gilead.com (G.N.); Mehtap.Guendogdu@gilead.com (M.G.); anika.moerner@gilead.com (A.M.); 3 Department of Hepatology and Gastroenterology, Charité Universitätsmedizin Berlin, Campus Virchow-Klinikum and Campus Charité Mitte, 10117 Berlin, Germany

**Keywords:** HCV therapy, PWID, opiate substitution, drug–drug interaction, direct-acting antivirals

## Abstract

People who inject drugs (PWID) are often affected by physical and psychological diseases and prone to co-medication. In Germany, about 50% of PWID are on opioid substitution therapy (OST). Comprehensive data on pharmacotherapy in these patients may help to select antiviral therapy against hepatitis C virus (HCV) infections and avoid drug–drug interactions (DDIs). We compared co-medication profiles based on statutory health insurance prescriptions (IQVIA database) of PWID (n = 16,693), OST (n = 95,023) and treated HCV patients (n = 7886). Potential DDIs with the most widely used HCV direct-acting agents (Sofosbuvir/Velpatasvir, Glecaprevir/Pibrentasvir and Elbasvir/Grazoprevir) were evaluated based on the Liverpool DDI database. Co-medication was present in 57% of PWID, 57% of OST, 44% of patients on HCV therapy and 46% in a subgroup receiving OST+HCV therapy (n = 747 of 1613). For all groups, co-medication belonging to ATC-class N (nervous system) was most commonly prescribed (in 75%, 68%, 41% and 62% of patients, respectively). Contraindications (i.e., DDIs precluding HCV therapy) were infrequent (0.4–2.5% of co-medications); potential DDIs with HCV therapies were shown for 13–19% of co-medications, namely for specific substances including some analgesics, antipsychotics, anticoagulants, lipid lowering drugs and steroids. In conclusion, concomitant pharmacotherapy is common and clinically relevant when treating HCV infection in PWID.

## 1. Introduction 

The prevalence of chronic hepatitis C among people who inject drugs (PWID) in Germany was found to be between 42% and 75% depending on the region in the cross-sectional DRUCK Study in 2015 [1,2]. The Robert Koch Institute estimates a prevalence of hepatitis C-antibodies of 68% among PWID [3]. To achieve the WHO’s proclaimed goal of hepatitis C virus (HCV) elimination by 2030, the special needs of this group have to be recognized. Much shorter duration of treatment compared to prior options, coupled with fewer side effects and easy oral application of direct acting antivirals (DAAs), has facilitated and improved HCV therapy considerably. German guidelines acknowledge the need and feasibility of treating PWID, especially in the context of substitution therapy and under close monitoring [4]. People on opioid substitution therapy (OST) can be effectively cured from HCV infection, as shown in several transnational studies [5,6,7,8,9,10]. Data from the German Hepatitis C Registry support these findings, with 95.1% of OST patients achieving sustained virological response with currently recommended, interferon-free treatment regimen [11]. A low-threshold approach to HCV treatment, provided by a trusted healthcare provider, might be working best to ensure optimal adherence to therapy [12].

Since PWID and patients on OST are often affected by physical and mental health disorders, polypharmacy is common to maintain the well-being of patients, but poses a challenge for possible drug–drug interactions with DAAs. Comprehensive data on the pharmacotherapy of these patient groups are scarce. In our analysis, the co-medication profiles of PWID and OST patient groups were examined with regard to possible interactions with DAAs that are recommended by current guidelines [13]. In comparison, the interaction profiles in a cohort of patients treated with one of the three currently recommended HCV DAAs, i.e., Sofosbuvir/Velpatasvir, Glecaprevir/Pibrentasvir or Elbasvir/Grazoprevir, were also analyzed. The same criteria were applied to a subgroup of HCV patients who were under substitution therapy during the HCV therapy. The aim was to provide a better understanding of the co-medication profiles of our target groups of PWID and OST patients and consequently to enable physicians to avoid possible interactions and optimize HCV therapy for these patients.

## 2. Methods

The data for this analysis were obtained using the IMS^®^ LRx database (IQVIA), which covers about 80% of all statutory health insurance prescriptions in Germany. This database provides anonymized prescription information from all fields of primary and specialty care, as well as private practices and outpatient hospital clinics. For the patients, it includes anonymized identification number, age, gender, insurance company and area of residence, as well as prescription information, an anonymized identification number for the prescriber, the date and package size. 

The observation period was October 2017 to September 2019, with a moving annual target (MAT) in September 2019 for patients on OST or HCV therapy. The MAT is used as a rolling yearly sum of all prescriptions over the course of the preceding 12 months.

For three patient cohorts and a subgroup of one of the patient’s cohort, all co-medications were compiled for the defined periods, and, in a second step, they were examined for potential drug–drug interactions (DDIs) with regard to state-of-the art direct acting antivirals (DAAs) in HCV therapy. Described in more detail below, these cohorts were categorized as OST cohort, PWID (without OST), HCV treatment cohort (Tx cohort) and, a subgroup of the latter, as the HCV treatment cohort with OST (HCV Tx/wOST cohort).

Co-medications of each cohort were categorized on the basis of E*ph*MRA (European Pharmaceutical Market Research Association) Anatomical Classification (ATC1/3 Level) and as individual substances, restricted to ATC classes A-S. ATC classes T (Diagnostic agents) and V (Various) were excluded. DDIs of any of the prescribed co-medications to the three most common DAAs (Sofosbuvir/Velpatasvir (SOF/VEL), Glecaprevir/Pibrentasvir (GLE/PIB) and Elbasvir/Grazoprevir (EBR/GZR)) were analyzed using the Hepatitis Drug Interaction Database from the University of Liverpool [14]. Here, the degree of interaction potential is depicted in a color-coded system with green (= no clinically significant interaction expected), yellow (= potential interaction likely to be of weak intensity), amber (= potential interaction) and red (= contraindication/co-administration not recommended). 

According to the Liverpool interactions checker, the effects of DDIs may be summarized as follows: (i) an increase in plasma concentration of co-medication (↑ co-med); (ii) a decrease in plasma concentration of co-medication (↓ co-med, which, of note, is not relevant in combination with DAAs that have no inducing effects); (iii) an increase in plasma concentration of DAAs (↑ DAA); or (iv) a decrease in plasma concentration of DAAs (↓ DAA).

### 2.1. OST Cohort

The population for the drug-substitution cohort includes patients with ≥ 1 prescription (Rx) of the medications in ATC class “Drugs used in opioid dependence” (N7F, i.e., methadone and buprenorphine) within the MAT September 2019 (with the exception of naloxone and naltrexone). Single doses for levomethadone, buprenorphine and buprenorphine/naloxone as well as methadone preparations were also considered. All co-medications prescribed in MAT September 2019 were evaluated. However, OST patients were expected to have no HCV therapy in the same MAT to rule out possible co-medication adjustment due to the initiation of HCV therapy.

### 2.2. People Who Inject Drugs

It is not possible to identify PWID from this anonymized patient dataset directly. However, it can be assumed that people currently receiving opioid replacement therapy in Germany (see above for definition) had been injecting drugs *before* they started OST. Other opioid use disorders without intravenous use can be excluded for this group. Therefore, PWID encompass all patients who started opioid substitution therapy in the current MAT (18 October 2018 to 9 September 2019) and who had not received such treatment for at least one year before that. Co-medication in the 365 days prior to start of OST was included in this analysis, reaching back as far as October 2017.

### 2.3. HCV Tx Cohort 

This cohort includes all patients with ≥ 1 prescription for ATC class “Hepatitis Antivirals” (J5D3) within the MAT September 2019, i.e., from 18 October 2018 to 9 September 2019. If more than one episode of HCV therapy occurred, the episode with the longest duration was chosen for this analysis. Duration was generally calculated according to the summary of product characteristics. Three oral DAA combinations (SOF/VEL, GLE/PIB and EBR/GZR) comprise 95% of the prescriptions in this cohort; the remaining HCV medications of this class, such as Ledipasvir/Sofosbuvir, were grouped together under “others”. Subgroups were formed for SOF/VEL, GLE/PIB and EBR/GZR patients. All co-medications prescribed during the continuous HCV therapy episode were taken into account.

### 2.4. HCV Tx/wOST Subcohort

As not all HCV treated patients had an opioid use disorder, a subcohort was formed of those HCV Tx patients who also received opioid substitution therapy during their continuous HCV therapy episode. Again, the DAA combinations SOF/VEL, GLE/PIB and EBR/GZR were prescribed in the majority of these patients (97%) and were individually evaluated regarding their interaction potential.

## 3. Results

### 3.1. Study Population

**Figure 1:**
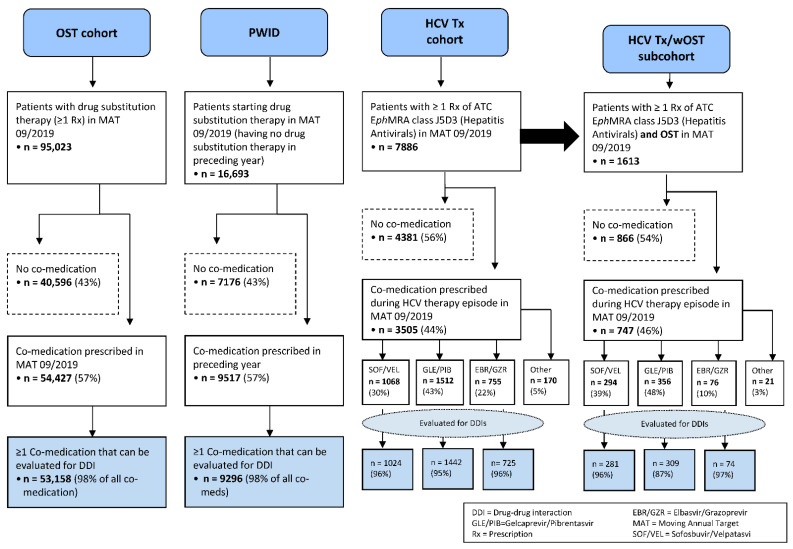
Consort Diagram (Patient disposition).

An overview of the study population is depicted in Figure 1. Of 16,693 PWID who started drug substitution therapy in MAT September 2019 and had no OST in the preceding year, n = 7176 (43%) received no co-medication, n = 9517 (57%) had one or more co-medications and n = 9296 had at least one co-medication that could be evaluated for potential DDIs (98% of co-medications). The median age for PWID with co-medication was 45 years (mean 48.1), 31.3% were women and 55.8% men (12.9% of unknown gender).

In MAT September 2019, n = 95,023 patients were identified with at least one prescription for opioid substitution therapy. In this cohort, n = 40,596 (43%) had no other medication. At least one co-medication was prescribed to n = 54,427 (57%) patients, of whom n = 53,158 received co-medications that could be evaluated for potential DDIs (98% of co-medications). The median age for the OST cohort with co-medication was 45 years (mean 46.3), 25.7% were women and 58.7% men (15.6% of unknown gender).

Regarding HCV therapy, n = 7886 patients received at least one prescription for the ATC class J5D3 (“Hepatitis Antivirals”) in MAT September 2019. For n = 4381 patients (56%), no co-medication was found. Overall, n = 3505 patients were identified as having at least one co-medication during their HCV therapy episode in MAT September 2019. The median age for patients with HCV therapy and at least one co-medication was 53 years (mean 53.1), 30.5% were women and 53.7% men (15.9% of unknown gender). 

Of the patients with HCV therapy, 95% could be stratified along the three major DAA regimes (SOF/VEL n = 1068 or 30%, GLE/PIB n = 1512 or 43% and EBR/GZR n = 755 or 22%), only n = 170 patients (5%) were treated with other hepatitis C antivirals, as well as co-medication. For the purposes of this analysis, only the three major subgroups were evaluated for potential DDIs to co-medication (SOF/VEL n = 1024, GLE/PIB n = 1442 and EBR/GZR n = 725 patients), covering 96%, 95% and 96% of all co-medications prescribed for these groups, respectively.

The HCV Tx/wOST subcohort consists of n = 1613 patients (20% of the n = 7886 HCV Tx patients) who received one or more prescriptions for opioid substitution therapy, as defined above, during their HCV therapy episode in MAT September 2019. In total, n = 747 patients (46%) received one or more co-medications during this episode. For these patients, the median age was 45 years (mean 44.8), 20.5% were women and 64.4% men (15.1% of unknown gender). The analysis once more focused on the major DAAs, which were prescribed in 97% of these patients, and subgroups were formed for n = 281 SOF/VEL patients, n = 309 GLE/PIB patients and n = 74 EBR/GZR patients, for whom the potential DDIs could be evaluated.

### 3.2. Co-Medication Profiles 

**Figure 2:**
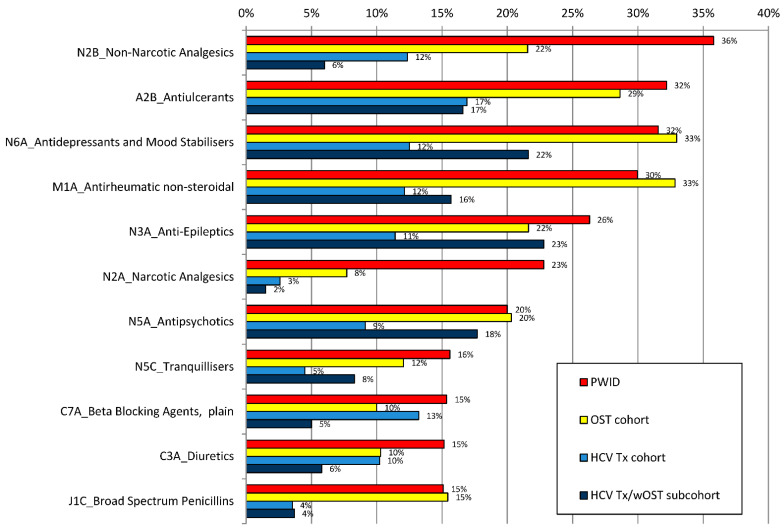
Most common co-medication classified by the E*ph*MRA (European Pharmaceutical Market Research Association) Anatomical Classification ATC3 level for three patient cohorts (people who inject drugs (PWID)), patients on opioid substitution therapy (OST cohort) and patients receiving HCV therapy (HCV Tx cohort)) and the subcohort with patients receiving HCV and OST therapy (HCV Tx/wOST subcohort).

Across all patient cohorts, medication of E*ph*MRA ATC class N (“Nervous System”) was by far the most common co-medication class on ATC level 1, with a patient number of n = 7186 drug PWID (75.5% of those with co-medication in this group), n = 37,125 patients with OST (68.2%), n = 1449 patients with HCV therapy (41.3%) and n = 460 patients with HCV Tx/wOST (61.6%). The second and third most frequent co-medications comprised ATC classes A (“Alimentary & metabolism”) and J (“General Anti-Infectives for systemic use”). The most common co-medications classified by ATC3 level for the four patient cohorts are shown in Figure 2. 

#### 3.2.1. PWID

For n = 9517 PWID with at least one co-medication, the most frequently prescribed ATC classes were non-narcotic analgesics (N2B), antiulcerants (A2B) and antidepressants and mood stabilizers (N6A) with more than 3000 patients in each class (35.8%, 32.2% and 31.6%, respectively). The most common co-medications of PWID on the substance level were metamizole sodium (N2B1) with a patient number of n = 2650 (27.8% of patients), pantoprazole (A2B2) with n = 2449 (25.7%), ibuprofen (M1A1) with n = 2156 (22.7%) and pregabalin (N3A0) with n = 1721 (18.1%).

#### 3.2.2. OST Cohort

In total, 54,427 OST patients received at least one co-medication. In this cohort, antidepressants and mood stabilizers (N6A) were first among the most frequently prescribed ATC classes with more than 15,000 patients each, followed by non-steroidal antirheumatics (M1A) and antiulcerants (A2B) (in 33.0%, 32.8% and 28.6% of patients, respectively). The most common co-medications for the OST cohort on the substance level were ibuprofen (M1A1) with n = 14,959 patient numbers (27.5% of patients), pantoprazole (A2B2) with n = 11,984 (22.0%), metamizole sodium (N2B1) with n = 9974 (18.3%) and pregabalin (N3A0) with n = 7173 (13.2%).

#### 3.2.3. HCV Tx Cohort (Patients Receiving HCV Therapy)

The most frequently prescribed ATC classes for the 3505 HCV Tx patients with at least one co-medication were antiulcerants (A2B) with 593 patients (i.e., 16.9% of the HCV Tx cohort), followed by beta-blocking agents (C7A) (13.2%), antidepressants and mood stabilizers (N6A) (12.5%), non-narcotic analgesics (N2B) (12.4%), non-steroidal antirheumatics (M1A) (12.1%) and anti-epileptics (N3A) (11.4%). On the substance level, pantoprazole (A2B2) was the most common with n = 428 patient numbers (12.2%), followed by metamizole sodium (N2B1) with n = 321 (9.2%) and ibuprofen (M1A1) with n = 317 (9.0%). While this corresponds largely to the other cohorts, some differences may be noted in the subgroups: ATC class J5D (hepatitis antivirals like ribavirin or hepatitis B therapies) is among the most common co-medications for the subgroup of HCV treated with SOF/VEL (n = 143 or 13.4% of the 1068 patients in this group), specifically ribavirin (J5D1) in n = 137 patients. Very likely, ribavirin has been prescribed as co-medication for cirrhosis patients during HCV therapy. In contrast to the other cohorts’ co-medication profiles, patients from the subgroup EBR/GZR received a larger share of co-medications from ATC class C (Cardiovascular System) with 44.1%; class N (Nervous System) took second place with a share of 35.9%. 

#### 3.2.4. HCV Tx/wOST Subcohort (Patients with HCV and OST Therapy)

The 747 patients in the subcohort HCV Tx/wOST with at least one other co-medication were most frequently prescribed substances of ATC classes anti-epileptics (N3A) (in 22.8% of patients), antidepressants and mood stabilizers (N6A) (21.6%), antipsychotics (N5A) (17.7%) and antiulcerants (A2B) (16.6%). The proportions in these first three classes are comparable to those of patients in the OST cohort. Pregabalin (N3A0) was the single most frequent substance, in n = 109 patients (14.6%). Of note, non-narcotic analgesics (N2B) played a lesser role than in the total HCV Tx cohort, 6.0% vs. 12.4% of all patients with HCV therapy. The same holds true for narcotics (N2A) with 1.5% vs. 2.6% and corticosteroids (H2A) with 1.2% vs. 2.8%. Fewer patients of the HCV Tx/wOST subgroup received beta-blockers (C7A) (5.0% vs. 13.2%) or diuretics (C3A) (5.8% vs. 10.2% in the overall HCV Tx cohort).

### 3.3. Potential DDIs with HCV Therapy in PWID and OST Patients

**Figure 3:**
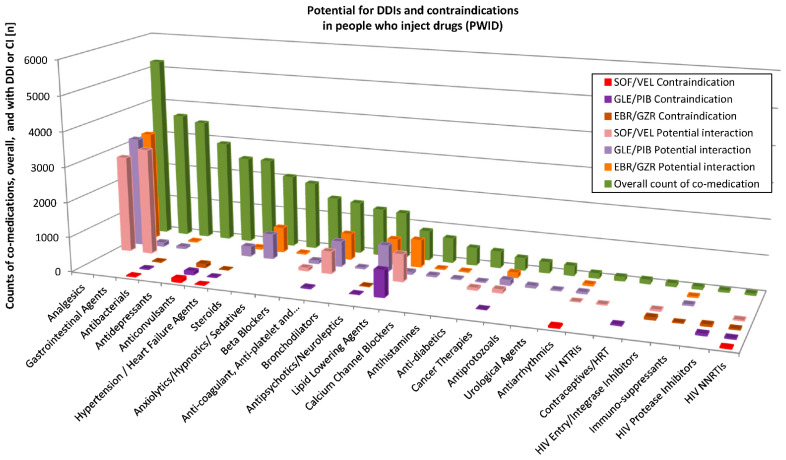
Potential for drug interactions between co-medication and SOF/VEL, GLE/PIB and EBR/GZR in people who inject drugs (PWID). For grouping of co-medication, the Liverpool DDI (drug–drug interactions) database was used.

**Figure 4:**
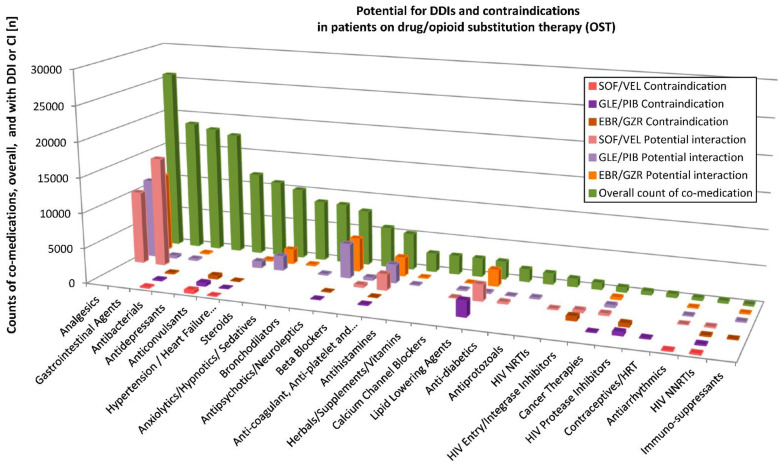
Potential for drug interactions between co-medication and SOF/VEL, GLE/PIB and EBR/GZR in the OST cohort. For grouping of co-medication, the Liverpool DDI (drug–drug interactions) database was used.

Potential DDIs with the three HCV DAAs were assessed for individual substances of the diverse ATC classes using the Liverpool database (http://www.hep-druginteractions.org, accessed on 13 December 2019). Detailed results for PWID and the OST patients are depicted in Figure 3 and Figure 4, respectively. For a patient with many various substances of a substance class (according to Liverpool database), only those with the highest interaction potential were considered. 

For all prescriptions across the drug classes in PWID (n = 39,461 counts of co-medications), they showed no interaction potential to SOF/VEL in n = 31,566 (79.9%), a weak interaction potential in n = 159 (0.4%), potential interaction in n = 7568 (19.2%) and were contraindicated together with SOF/VEL in n = 168 (0.4%) of the cases. When allocated to GLE/PIB, there was no interaction potential in n = 28,407 (72% of n = 39,461), a weak interaction potential in n = 3740 (9.5%), a potential interaction in n = 6332 (16%) instances and co-medication was contraindicated with GLE/PIB in n = 982 cases (2.5%). To EBR/GZR, no interaction potential was found in n = 32,014 (81.1% of 39,487 prescriptions), a weak interaction potential in n = 768 (1.9%), potential interaction in n = 6463 (16.4%) and co-medication was contraindicated with EBR/GZR in n = 242 (0.6%) counts.

In the OST cohort, n = 183,522 prescriptions were compiled across the drug classes that could be evaluated for possible interactions with SOF/VEL. Of these, n = 150,436 counts of co-medications (81.9%) showed no interaction potential. A weak interaction was possible for n = 550 (0.3%) and a potential interaction for n = 31,828 (17.3%). Overall, n = 738 (0.4%) of the co-medication counts were contraindicated. For GLE/PIB, no interaction potential was found in n = 137,935 (75.1%) of the 183,522 prescriptions, a weak interaction was possible for n = 18,461 (10.1%), a potential interaction for n = 23,871 (12.9%) and contraindication in n = 3485 (1.9%). Regarding EBR/GZR, no interaction potential was found for 155,866 counts of co-medications (84.9% of 183,610 prescriptions), weak interaction in n = 2096 (1.1%), a potential interaction in n = 23,892 (13.0%) and contraindication in n = 1756 (0.9%).

#### Prescribed Substances and Effects of DDIs

Among the most frequently prescribed substances, the following are associated with a potential interaction risk in combination with HCV DAAs: metamizole (↓ DAAs), pantoprazole (↓ DAAs, in particular SOF/VEL), omeprazole (↓ DAAs, in particular with SOF/VEL), rivaroxaban and apixaban (anticoagulants; both associated with an increase ↑ when co-administered with any of the three DAA combinations), simvastatin (↑) and atorvastatin (↑). 

Among all prescribed substances (in any of the cohorts), contraindicated or not recommended with the use of any of the three DAA combinations are carbamazepine (↓ DAAs) and oxcarbazepine (↓ DAAs) among anticonvulsants; the antibiotic rifampicin (↓ DAAs); and nevirapine (↓ DAAs), etravirine (↓ DAAs) and efavirenz (↓ DAAs) among non-nucleos/tide reverse transcriptase inhibitors (NNRTIs) in HIV treatment.

Of prescribed substances, the following were contraindicated or not recommended with one of the DAA regimens. For SOF/VEL, the antiarrhythmic amiodarone is among the contraindicated/not recommended drugs (based on reports of severe events such as bradycardia and heart block when the combination of SOF plus another DAA was used with concomitant amiodarone; the effect on amiodarone, velpatasvir and sofosbuvir concentrations are unknown). 

For GLE/PIB, simvastatin (↑) and atorvastatin (↑), as well as all HIV protease inhibitors employed (foremost darunavir used as part of the single tablet combination regimen darunavir/cobicistat/tenofovir alafenamide/emtricitabine and ritonavir) (↑) are among the contraindicated/not recommended substances. 

Contraindicated to GLE/PIB are ethinylestradiol-containing contraceptives (such as the combination levonorgestrel/ethinylestradiol (↑)) and the anticoagulant dabigatran (↑). With the use of GLE/PIB or EBR/GZR, the antipsychotic quetiapine (↑) presents a potential interaction risk, as well as dexamethasone (↑) and some antihypertensive drugs such as enalapril (GLE/PIB) (↑), eplerenone (↑), (EBR/GZR) and ranolazine (EBR/GZR) (↑). HIV protease inhibitors are contraindicated with EBR/GZR (↑).

### 3.4. DDIs in Patients Receiving HCV Therapy: SOF/VEL 

For the 1024 patients in the subgroup that was treated with SOF/VEL, this analysis considered 2520 prescriptions of co-medications for possible interactions with the hepatitis DAA (multiple entries per patient were possible). No interaction risk was found for 2107 (83.6%), and only weak interaction was found for eight counts of co-medication (0.3%). Potential interaction was identified for 404 counts (16%), and a contraindication was found in only one case (0.03%). This occurred in the class of anticonvulsants with the substance carbamazepine. 

When looking at the HCV Tx/wOST subgroup treated with SOF/VEL, 603 prescriptions were evaluated, of which 86.0% or n = 519 presented no interaction risk, while n = 84 counts (13.9%) had a potential for DDI, notably pantoprazole in n = 35 and metamizole in n = 16. No contraindicated co-medications were found.

ATC classes with potential interaction risks according to Liverpool database were analgesics, anticoagulants, beta-blockers, gastrointestinal agents, lipid lowering agents and among HIV antivirals the class of nucleos/tide reverse transcriptase inhibitors. Usually, only one or a few substances of the class were at risk, for example, among the beta-blockers, only carvedilol showed interaction potential to SOF/VEL; in the class of analgesics, only metamizole; and among HIV NRTIs, tenofovir disoproxil fumarate. Conversely, most lipid lowering agents do pose an interaction risk, with the one exception of pravastatin.

For co-medications in therapeutic classes of antibiotics, antipsychotics, hypertension/heart failure agents, anxiolytics/hypnotics/sedatives, bronchodilators, immunosuppressants and steroids, no interaction potential was established to the SOF/VEL subgroup.

### 3.5. DDIs in Patients Receiving HCV Therapy: GLE/PIB 

The GLE/PIB subgroup of 1442 patients included 3170 counts of co-medications prescribed during the HCV therapy episode (again, multiple entries per patient were possible). No interaction risk to GLE/PIB was found in n = 2452 (77.4%) and only a weak interaction potential for n = 354 (11.2%). An interaction potential was found in n = 334 patient counts (10.5%), and n = 30 (0.9%) were contraindicated. Contraindicated co-medications according to Liverpool database arose from the lipid lowering agents, namely atorvastatin and simvastatin. The HIV protease inhibitor-based combination darunavir/cobicistat/tenofovir alafenamide/emtricitabine is among the not recommended drugs. There was one instance of oxcarbazepine, which is not recommended, in the class of anticonvulsants, one in contraceptives (oral levonorgestrel/ethinylestradiol) and one in cancer therapies (vincristine).)

In the subgroup with OST, among the patients receiving GLE/PIB, n = 691 counts of co-medications could be evaluated. Only 8.4% of these (n = 58) posed a potential interaction risk, and three counts (0.4%) were contraindicated, all in the class of lipid lowering agents, with the use of atorvastatin.

Similar to SOF/VEL, the potential interaction to GLE/PIB was limited to one or few substances in the drug classes analgesics (here, only metamizole), antipsychotics (primarily quetiapine), beta-blockers (carvedilol), calcium channel blockers, gastrointestinal agents, hypertension/heart failure agents, immunosuppressants, steroids (dexamethasone) and anticoagulants (apixaban, edoxaban, phenprocoumon, rivaroxaban and warfarin).

The frequently prescribed drug classes antibiotics and gastrointestinal agents presented no or very little interaction potential in the GLE/PIB group. No interaction risk was found in antidepressants or anxiolytics/hypnotics/sedatives.

### 3.6. DDIs in Patients Receiving HCV Therapy: EBR/GZR

For the 725 patients in the EBR/GZR subgroup, n = 1847 counts of co-medications were evaluated for DDIs according to Liverpool database. Of these, n = 1584 (85.8%) had no interaction potential to the HCV DAA and n = 47 (2.5%) only a weak interaction potential, while n = 190 (10.3%) prescriptions showed a potential for interaction and 26 (1.4%) were contraindicated. 

Contraindicated or not recommended co-medications were found in anticonvulsants (carbamazepine, oxcarbazepine and phenytoin), immunosuppressants (one count of cyclosporine), anticoagulants (one count of dabigatran), boosted HIV integrase inhibitor regimens (namely, elvitegravir/cobicistat/tenofovir alafenamide/emtricitabine or elvitegravir/cobicistat/tenofovir disoproxil fumarate/emtricitabine), HIV protease inhibitors (darunavir/cobicistat/tenofovir alafenamide/emtricitabine and ritonavir) and HIV NNRTIs (nevirapine). Potential interactions included analgesics (here, metamizole), several anticoagulants (apixaban, edoxaban, phenprocoumon, rivaroxaban, tricagrelor and warfarin), antipsychotics (quetiapine), lipid lowering agents (simvastatin, atorvastatin, fluvastatin and rosuvastatin) and steroids (dexamethasone).

In the subgroup of HCV Tx/wOST patients on EBR/GZR therapy, of n = 150 prescriptions, no contraindicated substances were found, and only n = 10 (6.7%) presented a potential for interaction, namely quetiapine and metamizole. 

Frequently prescribed co-medications from antibiotics, antidepressants, anxiolytics/hypnotics/sedatives, beta-blockers and, in this case, gastrointestinal agents showed no or very little DDI potential to EBR/GZR.

## 4. Conclusions

In this analysis of statutory health insurance prescriptions, we observed differences in co-medication and the corresponding DDI profiles in the groups of PWID, patients on OST and treated HCV-infected individuals with or without OST. The use of co-medication was generally less frequent in treated HCV-infected individuals than in the other groups (44% vs. 57%); differences between PWID and OST patients were mainly seen for the use non-narcotic analgesics, in particular the use of metamizole. A considerable number of potential DDIs was found between the prescribed co-medications and the three DAA combinations that constituted 95% of all HCV antivirals (SOF/VEL, GLE/PIB and EBR/GZR). DDIs have been classified according to their specific effects on drug levels, namely an increase of either co-medication or DAAs levels implying a safety/tolerability risk or a decrease in DAA levels possibly associated with a reduction of efficacy. In PWID and OST patients, between 12.9% and 19.2% of the substances were identified to constitute a potential interaction risk, but only 0.4–2.5% were contraindicated. When looking at the actual co-medications prescribed during an HCV therapy episode, it was found that 10-16% of the co-medications were associated with a potential risk of DDIs (7–14% of co-medications in the HCV Tx/wOST subcohort), and there was only marginal appearance of contraindicated substances (e.g., HIV therapeutics, leading to an increase or decrease of DAAs). Statins, especially simvastatin and atorvastatin (the plasma concentrations of which increase in combination with DAAs), were prescribed to a much lesser degree than in PWID or OST patients, and the same was true for amiodarone. Carbamazepine, which is contraindicated, was prescribed only once. Antibacterials, although playing a large role in overall prescriptions, were seen much less in the HCV Tx subcohorts, and rifampicin not at all. 

The DDI potential was generally limited to single or few substances of a class, for example metamizole in analgesics (prescribed in 27.8% of PWID and 18.3% of OST patients). Metamizole is not listed as a drug with DDI potential in the summary of product characteristics; however, the University of Liverpool hepatitis drug checker describes a possibility of decreased concentration of SOF/VEL, GLE/PIB or EBR/GZR due to induction of CYP3A4 by metamizole. Pantoprazole is another substance to monitor among those most frequently prescribed (in 25.7% of PWID and 22% of OST patients). The summary of product characteristics of SOF/VEL cautions against concomitant use of omeprazole or other proton pump inhibitors at comparable doses; however, if necessary, administration may be spaced 4 h apart to avoid interaction [15]. Quetiapine (in antipsychotics) is the only other one among the top 20 prescribed substances with a potential interaction to GLE/PIB and EBR/GZR. 

If only one or two members of a drug class pose a threat of DDIs to HCV DAAs, e.g., quetiapine, carvedilol in beta-blockers or dexamethasone in steroids, these interactions can usually be avoided by switching to an alternative substance (which for some drugs has to be planned in advance) or, when possible, by interrupting use (e.g., for proton pump inhibitors or statins) during the weeks of HCV therapy. This seems to have been the case in some of the co-medications (i.e., carvedilol, rifampicin or carbamazepine) in our HCV Tx subcohorts and has been reported from other real-world observations [16]. 

Overall, the polypharmacy of PWID and OST patients was manifest along the expected therapeutic classes of analgesics, anti-inflammatory and antiepileptic drugs and antidepressants, together with the common proton pump inhibitors, propulsives, diuretics and ACE inhibitors. The large share in the ATC class J “Anti-infectives for systemic use” (in 38% of PWID, 36% of OST and 22% of HCV Tx patients), which includes beta-lactam antibiotics, may be explained with the primary or acute reason for seeking health care. A limitation of this anonymous dataset is the missing information about comorbidities, which may have shed more light on the need for medications. The Robert Koch Institute reports a much higher proportion of men among the hepatitis C patients in Germany (9.9 per 100,000 persons vs. 4.3 women per 100,000 persons), and the highest incidence in the age bracket 30–49 years [2], and official German statistics report a proportion of 22% female and 78% male opioid users, with a mean age of 39 years [17]. In our dataset, the proportion of women was between 20% (HCV Tx/wOST subcohort) and 31% (PWID). In terms of age, slight differences were observed for the HCV Tx cohort (with a slightly higher median age; 53 years) in comparison to the three other groups, namely the HCV Tx/wOST subcohort, the OST cohort and PWID (all with a median age of 45 years). Since we focused only on people who had at least one co-medication, there is a bias towards older age groups in our cohorts.

Another limitation is that the characteristics of the HCV Tx cohort in our dataset, including one-fifth HCV Tx/wOST, may differ from PWID and the OST patients in terms of concomitant use of recreational and illegal drugs. Moreover, there is a lack of information about concomitant use of over-the-counter drugs, alcohol, illegal or recreational drugs received via private prescription. Moreover, for females of childbearing age, the use of ethinylestradiol-containing contraceptives, contraindicated in combination with GLE/PIB, may be underestimated in our cohort since these are usually not reimbursed by the statutory health insurance and are therefore mainly issued on private prescription. However, although drug abuse presents a considerable health issue and psychosocial burden, the pharmacokinetics of most illicit drugs have no or hardly any interaction potential with OST (methadone and buprenorphine) or HCV antivirals, as shown in a recent large literature review [18]. Neither efficacy nor safety was expected to suffer with a concomitant use of opioids, stimulants or cannabinoids. As to the HCV DAAs, interaction was supposed to be very limited, since none of the abused substances are major inhibitors or inducers. Apart from the danger of side effects and a possible detrimental influence on adherence, recreational drug use should not constitute an obstacle to seeking HCV elimination. Moreover, the authors from several studies concluded that PWID can achieve SVR at high levels, ranging from 85% to 94%. In a German analysis [19], 85% of OST patients, 86% of non-OST/drug users and 92% of non-OST/non-drug patients reached SVR. Pooled data from studies on GLE/PIB [7], EBR/GZR [10] and the SIMPLIFY study investigating treatment with SOF/VEL [6] underscore the safety and efficacy of the HCV DAAs. In a recent meta-analysis on 5552 patients from 12 global cohorts receiving SOF/VEL, 680 of 689 (98.7%) patients with former or current intravenous drug use achieved SVR, increasing to 99.4% with no drug use [9]. 

These encouraging findings, together with the very manageable DDI potential for state-of-the-art HCV therapy, should help healthcare providers in making pro-active decisions for the hepatitis C treatment of PWID and OST patients. It is necessary to step up the pace, from the 61 per 100 PWID treated in 2017 in Western Europe [20], to reach the WHO’s 80% goal of HCV elimination by the year 2030. One potential approach would be to broaden the access to treatment to clinics and practices where PWID feel well cared for and welcome. The current guidelines for the treatment of HCV in Germany stress the advantages of a simultaneous initiation to OST therapy when starting HCV treatment [13], both for a close monitoring of the patients’ well-being and to strengthen patient–specialist relationship. The ANCHOR study in a Washington treatment center confirmed the merit of concurrent OST initiation with SOF/VEL treatment for 12 weeks [21]. Moreover, since PWID are known to attempt self-medication without physician’s knowledge or advise by abusing different substances to manage symptoms such as pain, depression or anxiety, physicians are expected to be highly aware of the problem, especially in order to choose a treatment regimen that has as few interactions as possible. Hopefully, given the excellent prospects of hepatitis C cure with modern antivirals, such as DAAs, and manageable drug–drug interactions, the goal of eradication may yet be reached.

## Data Availability

Not applicable.
